# Th17 Cells and IL-17 As Novel Immune Targets in Ovarian Cancer Therapy

**DOI:** 10.1155/2020/8797683

**Published:** 2020-02-21

**Authors:** Monika Bilska, Anna Pawłowska, Ewelina Zakrzewska, Agata Chudzik, Dorota Suszczyk, Marek Gogacz, Iwona Wertel

**Affiliations:** ^1^The First Department of Oncological Gynecology and Gynecology in Independent Public Teaching Hospital No. 1 in Lublin (Poland), Staszica 16, 20-081 Lublin, Poland; ^2^Independent Laboratory of Cancer Diagnostics and Immunology, The First Department of Gynecologic Oncology and Gynaecology, Medical University of Lublin, Staszica 16, Lublin 20-081, Poland; ^3^The First Medical Diagnostic Laboratory, St Joh's Cancer Center, Jaczewskiego 7, Lublin 20-090, Poland; ^4^II Department of Gynecology, Medical University of Lublin, Jaczewskiego 8, 20-954 Lublin, Poland

## Abstract

Ovarian cancer (OC) is usually diagnosed at an advanced stage and is related with poor prognosis. Despite numerous studies, the pathogenesis of OC is still unknown. Recent studies indicate the role of the immune system in the development and spread of OC. The identification of factors and mechanisms involved in that process and their modulation is crucial for creating effective antitumor therapy. We investigated the potential role of Th17 cells in OC patients (*n* = 71) by analyzing the frequencies of Th17 cells in three different environments, i.e., peripheral blood (PB), peritoneal fluid (PF), and tissue (Th17 infiltrating cells), and the concentration of IL-17A in plasma and PF of patients in terms of their clinical and prognostic significance. Th17 cells were analyzed by flow cytometry as a percentage of CD4^+^ lymphocytes that expressed intracellular expression of IL-17A. The level of IL-17A in plasma and PF were determined by ELISA. Our results showed accumulation of Th17 cells among tumor-infiltrating CD4^+^ lymphocytes (*p* < 0.001 in relation to PB). Moreover, the percentage of Th17 cells in both PB and PF of OC patients was significantly lower than that in benign tumors group (*n* = 35). There were no significant differences in the percentage of Th17 cells in PB, PF, and tissue in relation to clinicopathological characteristics of OC patients and survival. The lower percentage of Th17 cells in the PB and PF of OC patients may promote evasion of host immune response by cancer cells. The concentration of IL-17A in plasma of OC patients was higher (*p* < 0.0001) than that in both benign tumors and control group (*n* = 10). The PF IL-17A level in OC patients was higher (*p* < 0.0001) than that in women with benign ovarian tumors, indicating its synthesis in OC microenvironment. Higher IL-17A level in PF is correlated with longer (median: 36.5 vs. 27 months) survival of OC patients.

## 1. Introduction

Ovarian cancer (OC) is usually diagnosed at an advanced stage of development, which translates into adverse effects of treatment. Despite numerous studies, the pathogenesis of OC is still unknown. The mechanisms of tumor escape from immune surveillance, tissue infiltration, and metastasis are not yet explained. Under normal conditions, the immune system produces cells with anticancer potential. However, the response they induce is suppressed in the tumor microenvironment (TME). Recent studies indicate the involvement of the immune system in the development and spread of ovarian cancer [1–5]. In patients with OC, tumor infiltration by tumor-associated macrophages (TAMs), regulatory T cells (Tregs), and myeloid-derived suppressor cells (MDSCs) is observed, which stimulate immunosuppression and angiogenesis through various mechanisms, thereby contributing to disease progression and metastasis [6]. Abnormal vasculature is a hallmark of most solid tumors. It facilitates escape from immune surveillance and impairs perfusion and the entry of drugs from the circulation, limiting their anticancer effect [1, 5].

Th17 cells are a subpopulation of T helper cells (CD4^+^) that show high expression of interleukin 17 (IL-17A). They were described for the first time in 2003 by Aggarwal et al. [7]. Th17 cells develop from naive T cells in the presence of IL-6 and TGF-*β*. Their differentiation is controlled by transcription factors such as retinoic acid-related orphan receptors (ROR*γ*t, ROR*α*) and signal transducer and activator of transcription (STAT3) [8–10], whereas the presence of IL-1, IL-6, and IL-23 conditions their migration [11]. In addition to IL-17A, Th17 lymphocytes secrete IL-17F, IL-6, IL-21, IL-22, CCL20, and TNF-*α* [12–15]. Studies to date indicate a pleiotropic activity of IL-17 [16, 17]. On the one hand, IL-17 exhibits anticancer activity; on the other hand, it can promote progression of cancer [18, 19].

It has been proved that Th17 lymphocytes intensify migration of neutrophils to inflammatory foci [20] and are involved in the pathogenesis of autoimmune and allergic diseases. An increased percentage of these cells has been found in patients with lupus erythematosus, psoriasis, multiple sclerosis, rheumatoid arthritis (RA), and bronchial asthma [14]. Chang et al. showed a higher percentage of Th17 cells and cytokines associated with their differentiation (IL-6, TGF-*β*) in the peritoneal fluid (PF) of patients with endometriosis. Interestingly, the percentage of IL-10-producing Th17 cells was higher in advanced stages of the disease [21]. It should be emphasized that the role of Th17 cells in the pathogenesis of malignant tumors is not yet fully understood. However, their presence has been demonstrated in many types of cancer, especially in colorectal cancer, breast cancer, lymphoma, prostate cancer, gastric cancer, melanoma, and hepatocellular carcinoma [22]. Few studies have reported an increased percentage of Th17 lymphocytes in patients with ovarian cancer [18, 23]. A study by Kato et al. suggested that interleukin 17 secreted by Th17 cells may stimulate tumor progression through proangiogenic effects [24]. Further researchers confirmed this suggestion, showing an increased percentage of CD4^+^/IL-17^+^ lymphocytes among cells infiltrating ovarian cancer [25]. Kryczek et al. demonstrated the presence of Th17 cells in peripheral blood (PB), in PF, and among tumor-infiltrating cells in patients with advanced ovarian cancer [18]. The chemokines CCL20, CCL17, CCL22, RANTES, macrophage migration inhibitory factor (MIF), MCP1, and CCL4, secreted by cancer cells, fibroblasts, and dendritic and myeloid cells, were responsible for their recruitment to the tumor microenvironment. Th17 lymphocytes, which are detected in malignant tumors, are characterized by increased expression of CXCR4 and CCR6 receptors, respectively, for the chemokine (C-X-C motif) ligand-12; CXCL12 (SDF-1α) and chemokine (C-C motif) ligand 20 (CCL20) [26]. The SDF-1α chemokine participates closely with VEGF in the induction of angiogenesis. VEGF has been shown to induce CXCR4 expression for CXCL12 on endothelial cells, thereby increasing their sensitivity to the SDF-1*α* signal, and, synergistically with CXCL12, to induce migration of endothelial cells to TME. SDF-1*α* intensifies VEGF-induced endothelial cell proliferation and–synergistically with VEGF–protects them against apoptosis [27]. Furthermore, CXCL12 may enhance tumor vascularization also by regulating cellular composition in its microenvironment. SDF-1*α* has been shown to increase the migration of plasmacytoid dendritic cells (pDCs) into the tumor microenvironment [27, 28]. Curiel et al. demonstrated that pDCs present in the PF of patients with ovarian cancer produced high levels of proangiogenic cytokines (IL-8, TNF-*α*) and stimulated the development of new tumor blood vessels [29].

Other researchers have shown that Th17 lymphocytes may exhibit features of stem-like cells, whose activity may be partially controlled by signalling pathways induced by hypoxia-inducible factors, especially by HIF-1 [30].

The anticancer activity of Th17 lymphocytes is associated with their effect on other cells of the immune system [18]. Th17 cells have been shown to increase migration of cytotoxic lymphocytes (CTL), NK cells, macrophages, and neutrophils. In addition, they stimulate maturation of DCs, resulting in increased expression of surface molecules of the major histocompatibility complex (MHC). Recruited cells interact through different mechanisms with cancer cells, leading to their death. Activation of cytotoxic lymphocytes by DCs presenting tumor antigens induces an anticancer response and may lead to inhibition of tumor growth, but data are ambiguous [31].

## 2. Aim of the Study

In this study, we investigated the potential role of Th17 cells in ovarian cancer patients by analyzing the frequencies of Th17 cells in three different environments, i.e., peripheral blood, peritoneal fluid and tissue (Th17 infiltrating cells) and the concentration of IL-17A in plasma and PF of patients in terms of their clinical and prognostic significance.

## 3. Materials and Methods

### 3.1. Study Population

A total of 71 patients with proven OC diagnosis were qualified to the research. The examined clinical materials were the drawing peripheral blood, peritoneal fluid, and tumor tissue. They were collected from patients that were operated from 2012 to 2017 at The First Department of Oncological Gynecology and Gynecology in Independent Public Teaching Hospital No. 1 in Lublin (Poland). The patients did not receive antitumor treatment before and drugs that might have influence on immune system. The exclusion criteria were also infections, allergy, and autoimmunological diseases. Patient characteristics at the time of OC diagnosis are presented in Table 1.

Moreover, the researches were conducted in the reference group of patients (*n* = 35) at the age 22–78 (median 28 years) with benign cyst of ovary (serous cyst of ovary) and in the control group of healthy women (*n* = 10) at the age 21–51 (median 29 years). The PB of control group were obtained thanks to Regional Centre of Blood Donation and Blood Treatment in Lublin. Every patient signed the written acquiescence to participation in the study. The conducted research received approval of Bioethics Committee at Medical University of Lublin (KE-0254/280/2015).

### 3.2. Cell Isolation

Peripheral blood samples were collected before the surgical procedure into heparinised tubes (Sarstedt, Germany) and immediately processed. Mononuclear cells (MNCs) were separated by density gradient centrifugation with Gradisol L (AquaMedica, Poland) for 20 minutes at 700 ×g at room temperature. Interphase cells were removed, washed twice, and resuspended in phosphate-buffered saline (PBS, PAA Laboratories GmbH, Austria).

Peritoneal fluid and tissue were collected aseptically during the operation. MNCs from PB/PF were isolated by density gradient centrifugation on Gradisol L (AquaMedica, Poland) for 20 minutes at 700 ×g at room temperature. For isolation of tumor-infiltrating MNCs, freshly resected tissue was minced, placed into a gentleMACS C tube, and processed using Tumor Dissociation Kit (MiltenyiBiotec). The resulting cell suspension was filtered through 70 mm meshfilted (BD Biosciences) and subjected to the density centrifugation as described above. Mononuclear cells were isolated within 2 h of draw and used for *in vitro* cell culture and flow cytometry analysis.

### 3.3. Intracellular IL-17A Staining

*In vitro* mononuclear cell cultures were used to analysis of Th17 cells subpopulation. Taking into account viability and MNCs concentration, intracellular IL-17A analysis was performed on fresh PB (*n* = 59), PF (*n* = 35) and tumor (*n* = 17) samples from ovarian cancer patients and on fresh PB and PF samples from the reference group (*n* = 32).

Mononuclear cells (2 × 10^6^ cells/ml) were cultured in RPMI 1640 supplemented with 2 mmol/l L-glutamine, 5% human albumin (ZLB Bioplasma, Bern, Switzerland), 100 U/ml penicillin, and 100 mg/ml streptomycin. Cells were stimulated with 25 ng/ml of Phorbol 12-Myristate 13-Acetate (PMA) and 1 mg/ml of ionomycin (Sigma, Germany) in the presence of BD GolgiStop (BD Pharmingen, USA) for 4 hours at 37°C in a 5% CO_2_ atmosphere. Cultured cells were washed twice in PBS, divided into tubes, and then stained with monoclonal antibodies (MoAb) against the cell-surface markers anty-CD3 FITC, anty-CD4 PerCP-Cy5.5 (BD Bioscience, USA). Following membrane staining, cells were fixed and permeabilized using IntraPrep Kit (Immunotech, France) according to the manufacturer's instructions. Cells were then intracellularly stained with anty-IL-17A PE (e-Bioscience, USA) or a PE mouse IgG1, қ isotype control (e-Bioscience, USA). Finally, cells were washed and the percentage of Th17 cells was analyzed by flow cytometry (FACSCanto I Becton Dickinson, USA). Th17 cells were analyzed as percentage of CD4^+^ that expressed intracellular expression of IL-17A.

**Figure 1:**
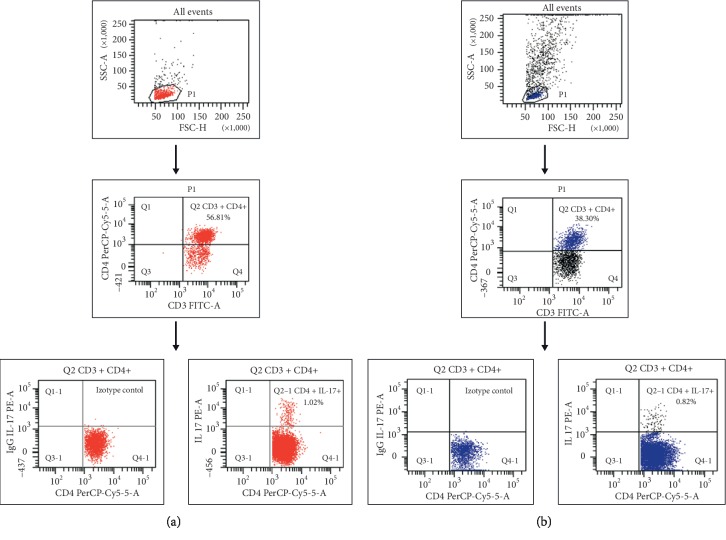
Flow cytometric analysis of Th17 cells in the peripheral blood (a) and tumor (b) of serous ovarian cancer patient.

For each analysis, 100,000 events were acquired and analyzed using FacsDiva software. Isotype-matched MoAb were used to verify the staining specificity and as a guide for setting the markers to delineate positive and negative populations. Dot plots, illustrating the analysis method for the identification of CD3^+^ CD4^+^IL-17^+^ T cells, are shown in Figure 1.

### 3.4. ELISA

Plasma/PF samples were rendered cell-free by centrifugation (1000 ×g/15 min) and the supernatants were stored at −80°C until the time of analysis. The concentrations of IL-17A in plasma and PF were determined by ELISA (enzyme linked immunosorbent assay) according to the manufacturer's instruction. Human IL-17A High sensitivity ELISA kit (Bender MedSystems, USA) and sensitivity of 0.01 pg/ml were used. Measurement was estimated through ELISA El × 800 reader (BIO-TEK Instruments, USA).

### 3.5. Statistical Analysis

The statistical analysis of results was conducted using Statistica 12.0 PL.

The Wilcoxon paired test was used to compare the results in PB, PF, and tissue. The Mann-Whitney *U* test was applied to the results of statistical comparison between the studied groups (Control/OC/Reference group). Relationships between two parameters were investigated using Spearman's rank correlation test. The probabilities of overall survival (OS) were estimated using the Kaplan–Meier method and differences in survival curves were calculated using the logrank test; *p* value less than 0.05 was considered statistically significant. The data are presented as medians, minimum, and maximum.

The dot plots show representative data from OC patients, illustrating the analysis method for identification of CD3^+^/CD4^+^/IL-17A^+^ (Th17) cells. An acquisition gate was established based on FSC and SSC that included mononuclear cells. A population P1 was drawn around the lymphocytes. Next, the P1 gated events were analyzed for CD3 FITC and CD4 PerCP-Cy5.5 staining and positive cells (CD3^+^/CD4^+^) were gated (region Q2). The final dot plots CD4 PerCP-Cy5.5 versus mouse IgG1 PE and CD4 PerCP-Cy5.5 versus IL-17A PE were established by combined gating of events using population P1 and region Q2. The number in the upper right quadrant in the region Q1-2 represents the percentage of CD3^+^/CD4^+^/IL-17A^+^ (Th17) cells.

## 4. Results

Accumulation of Th17 cells among ovarian cancer infiltrating cells in OC patients.

**Figure 2:**
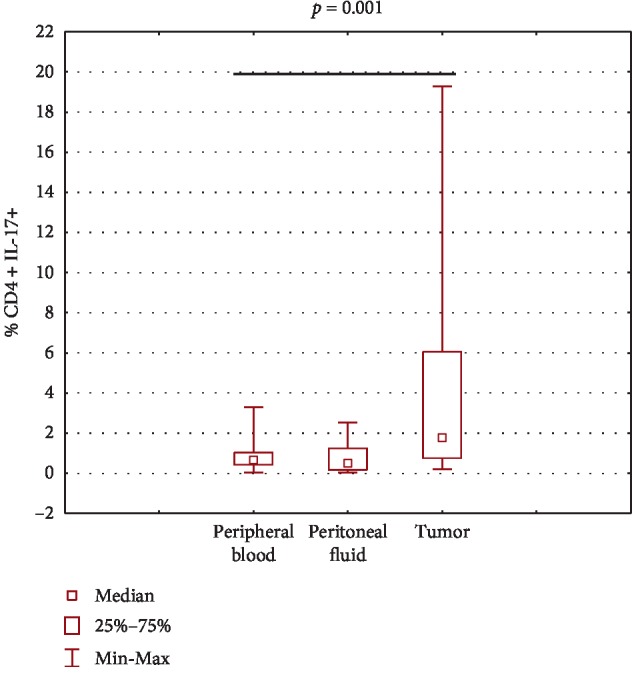
The percentage of Th17 cells in peripheral blood, in peritoneal fluid, and among ovarian cancer infiltrating cells.

The highest percentage of CD4^+^ lymphocytes expressing IL-17 was detected among CD4^+^ T cells infiltrating ovarian cancer and it was significantly higher (*p*=0.001) compared to PB. The percentage of CD4^+^IL-17^+^ cells was higher in the tumor than that in the peritoneal fluid; however, the difference did not reach statistical significance (*p*=0.08). There was no statistically significant difference (*p* > 0.05) in the percentage of CD4^+^IL-17^+^cells detected in peripheral blood and PF. The obtained results are presented in (Figure 2).

### 4.1. Clinical Significance of Th17 Cells in the Peripheral Blood, Peritoneal Fluid, and Tumor Tissue of OC Patients

To investigate the clinical potential of CD4^+^IL-17^+^ cells, we determined its association with the patients' clinicopathological characteristics. There was no statistically significant difference (*p* > 0.05) in the percentage of CD4^+^IL-17^+^ cells in PB, PF, and tumor depending on the FIGO stage, histological grade, and type of OC according to Kurman and Shih classification. There was also no statistically significant difference (*p* > 0.05) in the percentage of CD4^+^IL-17^+^ cells depending on the menopausal status of patients.

Relationship between the percentage of Th17 cells in PB, PF, and tumor and concentration of IL-17A and clinicopathological characteristics of OC patients are shown.

There was no significant correlation (*p* > 0.05) between the percentage of Th17 cells in the PB and the plasma IL-17A level and between the percentage of Th17 cells and concentration of IL-17A in the PF of ovarian cancer patients.

There was no significant correlation (*p* > 0.05) between the percentage of Th17 cells and FIGO stage, histological grade, and type of OC according to Kurman and Shih classification, menopausal status, and plasma Ca125 level.

The percentage of Th17 cells in peripheral blood and peritoneal fluid of patients with ovarian cancer and in the group with benign ovarian tumor is shown.

**Figure 3:**
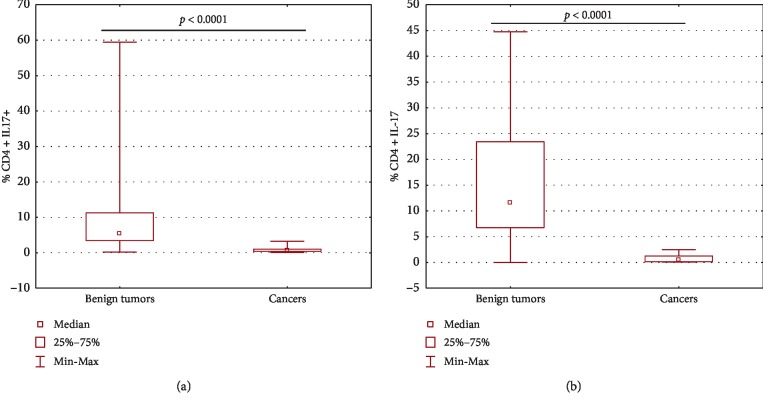
The percentage of Th17 cells in peripheral blood (a) and peritoneal fluid (b) of patients with ovarian cancer and in the group with benign ovarian tumor.

In both peripheral blood and peritoneal fluid, the percentage of CD4^+^ lymphocytes expressing IL-17 was significantly lower (*p* < 0.0001) in the ovarian cancer group than in the benign ovarian tumor group (Figure 3).

### 4.2. The Percentage of Th17 Cells in Peripheral Blood and Peritoneal Fluid of Patients with Ovarian Cancer or in the Group with Benign Ovarian Tumor

**Figure 4:**
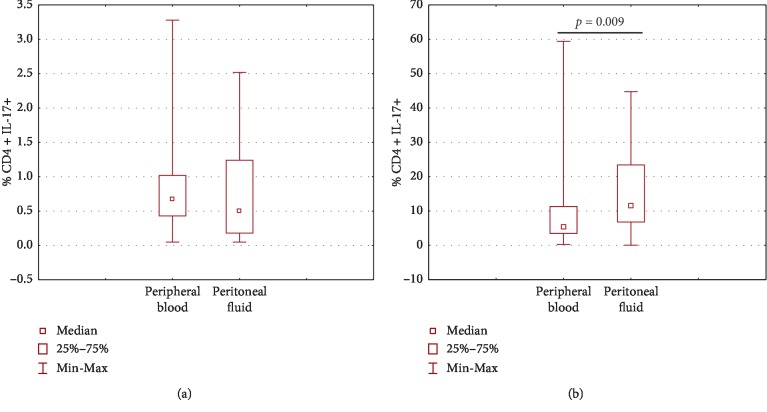
Comparison of Th17 cells percentage in PB and PF of ovarian cancer (a) and in the group with benign ovarian tumor (b).

The percentage of Th17 cells in OC patients was lower in PF than that in PB; however, the difference did not reach statistical significance. The percentage of Th17 cells in patients with benign tumors was significantly higher (*p*=0.009) in PF than that in PB (Figure 4).

### 4.3. The Analysis of Relationship between Th17 Cells Percentage and Survival of Ovarian Cancer Patients

In order to analyse relationship between Th17 cells and survival of ovarian cancer patients, the patients were classified depending on the examined factor values that were qualified as higher or lower than the median reached for the group of patients. The patients' survival was measured from the time of histopathological diagnosis to death or the end of observation time in living patients. In any of the examined microenvironments (PB, PF, or tumor), we have not demonstrated statistically significant relationships (*p* > 0.05) between the percentage of Th17 cells and five-year survival of ovarian cancer patients.

### 4.4. Accumulation of IL-17A in Plasma of Patients with Ovarian Cancer in Relation to the Group with Benign Ovarian Tumors and the Control Group

**Figure 5:**
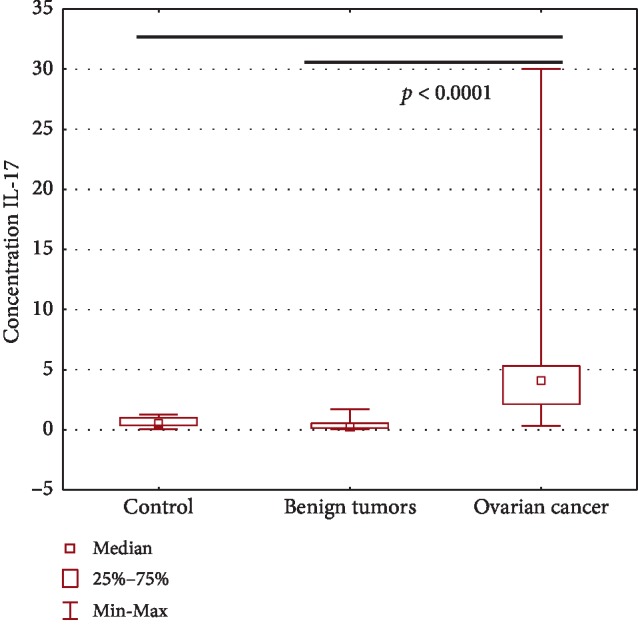
Plasma IL-17A concentration in patients with ovarian cancer, in the group with benign tumors and in the control group.

The highest concentration of IL-17A was detected in the plasma of ovarian cancer patients and it was significantly higher (*p* < 0.0001) compared to both the group of benign ovarian tumors and the control. The obtained results are presented in Table 2 and in (Figure 5).

**Figure 6:**
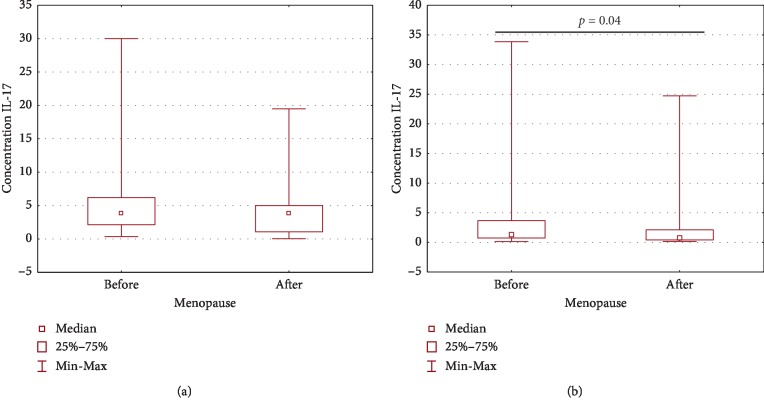
Plasma (a) and PF (b) IL-17A concentration in patients with ovarian cancer before and after menopause.

There was no statistically significant difference (*p* > 0.05) in both plasma and PF IL-17A concentration depending on FIGO stage, histological grade, and type of OC according to Kurman and Shih classification. There was also no statistically significant difference (*p* > 0.05) in plasma IL-17A concentration depending on the menopausal status of the patients. What is interesting, IL-17A concentration in the PF of premenopausal patients was significantly higher (*p*=0.04) than that in the postmenopausal group (Figure 6).

### 4.5. Relationship between the Concentration of IL-17A in Plasma and PF and Clinicopathological Characteristics of OC Patients

There was no significant correlation (*p* > 0.05) between the plasma level of IL-17A and FIGO stage, histological grade, and type of OC according to Kurman and Shih classification, menopausal status, and Ca125 level.

There was also no significant correlation (*p* > 0.05) between the PF level of IL-17A and FIGO stage, histological grade, and type of OC according to Kurman and Shih classification, and Ca125 level. What is interesting, in the PF, there was significant correlation (R Spearman −0.221, *t* (*N* − 2) −2.084, *p*=0.04) between the level of IL-17A and menopausal status of patients.

### 4.6. Comparison of Plasma and Peritoneal Fluid IL-17A Levels in Patients with Ovarian Cancer or Benign Ovarian Tumors

**Figure 7:**
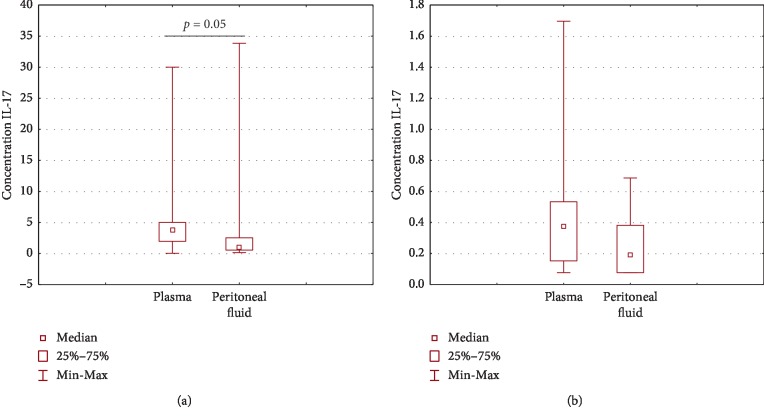
Comparison of plasma and PF IL-17A concentration in patients with ovarian cancer (a) or in the group with benign ovarian tumors (b).

The plasma IL-17A concentration was higher (*p*=0.05) compared to that detected in the PF of patients with ovarian cancer (Figure 7). The plasma IL-17A concentration in patients with benign ovarian tumors was higher than that in PF; however, the difference did not reach the level of statistical significance (*p* > 0.05).

### 4.7. Assessment of IL-17A Concentration in the Peritoneal Fluid of Patients with Ovarian Cancer and in the Group with Benign Ovarian Tumors

**Figure 8:**
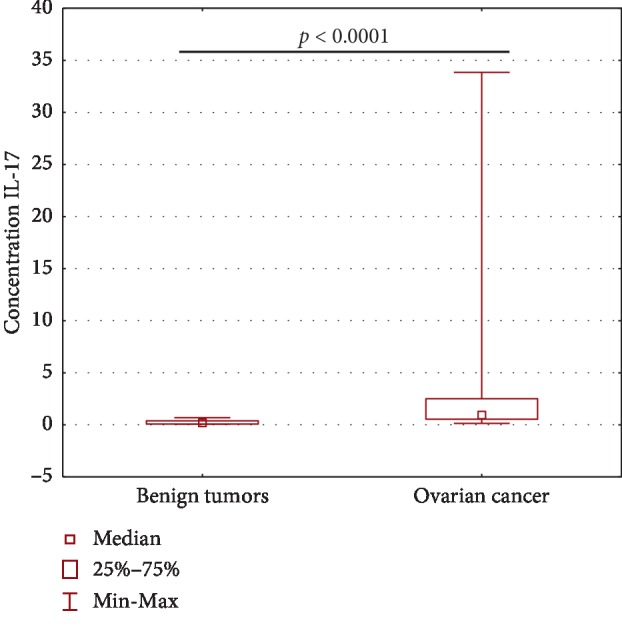
IL-17A concentration in the PF of patients with ovarian cancer and in the group with benign ovarian tumors.

The IL-17A concentration in the PF of ovarian cancer patients was significantly higher (*p* < 0.0001) compared to the group with benign ovarian tumors (Figure 8).

### 4.8. The Analysis of Relationship between Concentration of IL-17A in Plasma and PF and Survival of OC Patients

**Figure 9:**
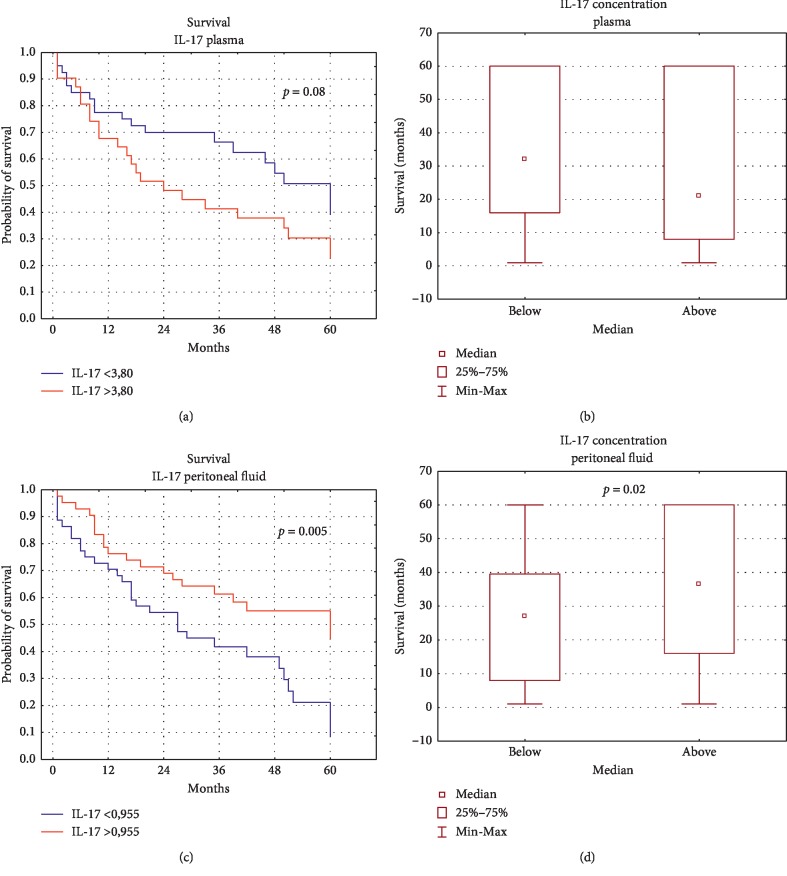
Relationship between the level of IL-17 in the plasma (a, b) and PF (c, d) and survival of OC patients.

**Figure 10:**
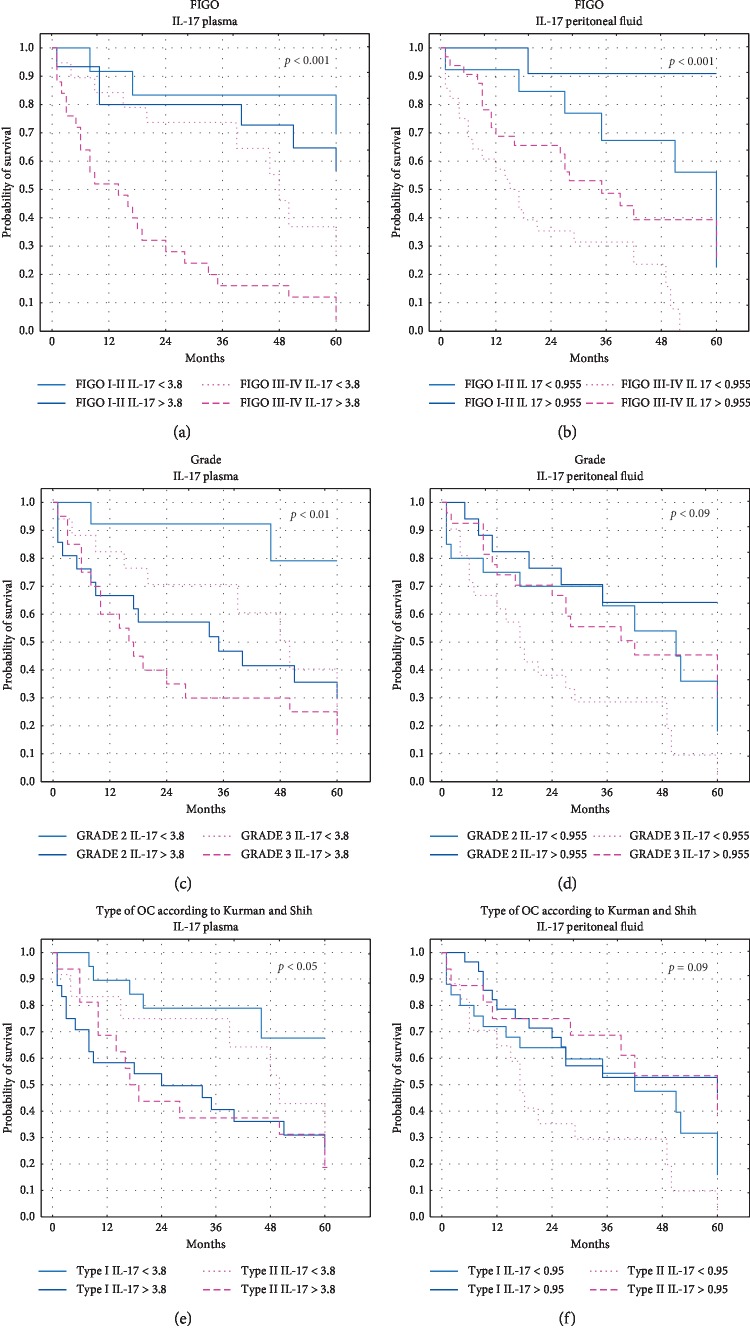
The relationship between IL-17 concentration, clinical parameters, and five-year survival of OC patients.

There was no statistically significant correlation between plasma IL-17A concentration and five-year survival of OC patients (*p* > 0.05). What is interesting, the survival of OC patients did not depend on IL-17A level in the peritoneal fluid and was significantly longer (the median: 36.5 months vs 27 months) in patients with higher IL-17A level than that in patients with lower IL-17A level in PF (Figure 9). The relationship between IL-17 concentration, clinical parameters, and five-year survival of OC patients is shown in Figure 10.

## 5. Discussion

In our study we evaluated the percentage and distribution of Th17 cells in ovarian cancer patients in three different environments, i.e., peripheral blood, peritoneal fluid, and tissue (Th17 infiltrating ovarian cancer), as well as the concentration of proinflammatory cytokine IL-17A in plasma and PF in terms of their clinical and prognostic significance.

It should be emphasized that the role of Th17 cells in ovarian cancer immunobiology is not clear. As a result of contact with cancer cells, they can produce both pro- and anticancer factors. According to few reports, the population of Th17 has proangiogenic properties [24, 32]. However, the mechanisms of this activity in ovarian tumors are still not fully understood. In addition, it should be noted that these issues are not widely addressed in the available literature.

Our own research showed differences in the percentage and distribution of Th17 cells in peripheral blood, peritoneal fluid, and the tumor. The highest percentage of CD4^+^ lymphocytes with intracellular expression of IL-17A was observed among CD4^+^ cells infiltrating OC and it was significantly higher than that in peripheral blood. Similarly, Kryczek et al. demonstrated an accumulation of Th17 cells among cells infiltrating ovarian cancer, compared to PB and lymph nodes [18]. Other researchers also showed a higher concentration of Th17 subpopulations in tissue in patients with OC, although the difference they found did not reach statistical significance [33]. The accumulation of Th17 cells in tumors observed in our research suggests the existence of factors increasing their migration and/or differentiation in the OC microenvironment. Kryczek et al. proved that Th17 cells infiltrating the tumor showed high expression of molecules such as CXCR4, CCR6, and CD161. They stated that these molecules may increase the migration of Th17 cells into the tumor microenvironment. Some *in vitro* studies also demonstrated the effect of other immune cell populations on inducing Th17 cell differentiation. Tumor-associated macrophages, which induced Th17 cells differentiation to the greatest extent, were particularly important in this respect. Further studies have shown that cytokines such as IL-1*β* and IL-23 are an important mediator of Th17 induction by TAMs in patients with ovarian cancer [18].

In our research, we showed a significantly lower percentage of Th17 cells in both PB and PF in ovarian cancer patients, compared to the group with benign ovarian tumor. The causes of the observed phenomenon are not fully understood. However, it suggests the existence of factors inhibiting the differentiation of Th17 cells in the examined female population. Other researchers have shown that Th17 cell differentiation in ovarian cancer patients can be inhibited by Tregs cell activity. It was observed in *in vitro* studies that Treg cells inhibited not only Th17 cell differentiation but also IL-17 synthesis by these cells. Kryczek et al. demonstrated that Treg cells isolated from OC express the CD39 molecule, an ectonucleotidase that converts ATP to adenosine, thereby participating in suppressing Th1 cell differentiation through the adenosinergic pathway [18]. Interestingly, Ye et al. noticed that Tregs and Th17 cells can transform each other and exhibit different functions in the tumor microenvironment. As a result of conversion into IFN-*γ*^+^FoxP3^+^ T lymphocytes, Th17 cells acquire strong immunosuppressive properties [34]. It is possible that this conversion is one of the mechanisms by which the cancer escapes from immune surveillance. IL-2 has been shown to regulate the balance between Tregs and Th17 cells in a tumor, reducing the percentage of Th17 cells in the TME and increasing the number of Tregs [35]. Other researchers have demonstrated that local administration of IL-2 increases the percentage of Th17 producing IFN-*γ* and TNF-*α* among tumor-infiltrating lymphocytes and induces the conversion of Tregs to Th17 [36].

Literature data suggest that Th17 cells induce synthesis of chemokines (CXCL9, CXCL10), which increase migration of effector cells that induce a Th1-type response in the OC microenvironment. It has been shown that tumor-infiltrating Th17 lymphocytes display low expression of activation markers and effector functions, such as HLA-DR, CD25, granzyme B, and perforin, and they do not mediate direct cytotoxic activity targeted against cancer cells. However, they recruit other immune system effector cells. According to this concept, IL-17 and IFN-*γ* secreted by Th17 synergistically induced synthesis of CXCL9 and CXCL10 chemokines, which in turn increased migration of effector T lymphocytes into the OC microenvironment. It was observed that the level of these chemokines correlated with the percentage of CD8^+^ and NK cells infiltrating ovarian cancer [18]. In addition, the Th17 subpopulation stimulates cancer cells to secrete CCL20, a chemokine that enhances migration of dendritic cells to the TME [37]. In this respect, Th17 cells may participate in the induction of anticancer response in patients with OC. The reduced percentage of Th17 cells in patients with ovarian cancer observed in our own studies may be one of the strategies by which cancer escapes from the immune system.

Another evidence of the protective, anticancer role of Th17 cells is the results obtained by Hirahara et al., who observed inhibition of OC growth in a hamster after implantation of IL-17-gene-transfected tumor cells [38]. Other reports show that immunotherapy based on inhibition of the activity of the enzyme indoleamine 2,3-dioxygenase (IDO) increases the anticancer activity of Th17 cells [39].

Contradictory results were obtained by Zhu et al., who compared the concentration of Th17 cells infiltrating ovarian cancer and benign tumors by confocal microscopy. Their results indicate a significantly higher percentage of Th17 cells in malignant ovarian tumors, compared to benign lesions, which suggests the involvement of Th17 in cancer progression [40].

Numerous studies emphasize the role of other subpopulations of tumor-infiltrating lymphocytes (TILs) in the induction of anticancer responses [41, 42]. They have been proved to be a prognostic factor in many types of cancer, including melanoma, colorectal cancer, and ovarian cancer [43–47]. Zhang et al. conducted a study on 186 patients with OC and showed that as many as 55% of women with CD3^+^ TILs achieve a 5-year survival rate of 38%, while only 4.5% of women without TILs achieve a similar level [48]. The importance of TILs in cancer is also confirmed by a meta-analysis by Hwang et al. of 10 clinical studies involving 1815 patients with OC. As a result, it was established that the presence of both CD3^+^ and CD8^+^ TILs is associated with increased long-term survival. It has also been shown that a high ratio of CD8^+^ TILs to FoxP3^+^ Treg cells in patients with OC is a positive prognostic factor [49]. Furthermore, a high percentage of CD8^+^ TILs has been shown to be a prognostic factor for longer survival in patients with low-differentiated ovarian cancer [50].

There are few studies in the available literature assessing the relationship between the percentage of Th17 cells and the survival of ovarian cancer patients. In our research, we found no statistically significant relationship between the percentage of Th17 cells and the survival of patients with OC in any of the examined environments. Similarly, Winkler et al. showed no significant relationship between the percentage of CD4^+^IL-17^+^ cells either infiltrating the tumor or those detected in peripheral blood and the survival time of the patients [33]. It should be emphasized that the results of the assessment of the described relationship in cancer patients are divergent. 18 of the 27 studies analysed by Punta et al. showed that Th17 cells infiltrating tumors were a negative prognostic factor. Interestingly, five studies showed a positive correlation between Th17 cells and survival, including in cervical cancer and recurrent ovarian cancer. In the remaining four analysed reports, no significant correlations were found between the assessed cells and survival, including in epithelial ovarian cancer [19].

It was interesting to observe in our study that there was a significantly higher concentration of IL-17A in both the plasma and PF of patients with ovarian cancer, compared to the group with benign ovarian tumors. In contrast, Wang et al. did not observe any statistically significant differences in the concentration of IL-17 in the plasma of ovarian cancer patients, compared to the group with benign ovarian tumors and the group of healthy women [51]. The results we obtained are consistent with the results of Malekzadeh et al. who showed a higher concentration of IL-17A in the serum of patients with papillary adenocarcinoma, compared to the control group. They also observed a higher concentration of the assessed cytokine in the serum of patients with OC with a low degree of histological differentiation, compared to cancer with a high and medium degree of differentiation. According to them, this observation may suggest the existence of various sources of IL-17 (Th17 or cancer cells) involved in the production of this cytokine at particular stages of tumor progression [52]. Tang et al. showed a higher level of IL-17 expression in the tissue of ovarian malignant tumors, compared to benign tumor tissue and controls. The level of IL-17 expression assessed in the study correlated with the stage of the disease according to FIGO and the degree of histological differentiation of the tumor [53].

The higher concentration of IL-17A in the peritoneal fluid of OC patients noted in our study may contribute to local dissemination of cancer by affecting the formation of peritoneal implants. In addition, IL-17 may induce local IL-6 synthesis in the peritoneal environment, increase macrophage infiltration into the TME, and induce inflammatory processes and angiogenesis. Kato et al. showed a proangiogenic role of IL-17 in their study on an ovarian cancer mouse model. They found a significantly higher average number of blood vessels within ovarian tumors that were characterized by increased IL-17 mRNA expression [24]. Numasaki et al. noted that IL-17 directly affects endothelial cells, induces the production of proangiogenic cytokines and lymphokines, and enhances their activity. They confirmed the results on a SCID mouse model with implanted human non-small cell lung cancer (NSCLC). Using the RT-PCR technique, they showed that high expression of IL-17 in the tissue of primary NSCLS is associated with increased vascularization of the tumor, and thus with worse prognosis and shorter survival [14]. Other researchers report that IL-17 stimulates synthesis of proangiogenic chemokines, i.e., CXCL1, CXCL5, and CXCL8. IL-17 has been shown to promote tumor progression in colorectal cancer, lung cancer, breast cancer, stomach cancer, hepatocellular carcinoma, and pancreatic cancer [54–58]. It has been found that IL-17 mediates inflammation and may induce the production of other inflammatory factors, including TNF-*α*, IL-6, and IL-1*β* [59]. In turn, secreted IL-6 activates STAT3, which increases the level of expression of genes inducing tumor progression and metastasis [36]. Tartour et al. observed that transfection of human cervical cancer cells with IL-17 potentiated tumor growth after transplanting them into mice. Interestingly, mice lacking IL-17 exhibited slowed growth of melanoma (B16) and bladder cancer (MB49), which suggests the role of IL-17 in promoting tumor development [60]. IL-17 has been shown to promote VEGF secretion, enhancing tumor angiogenesis, cancer cell invasion, and metastasis [61]. Recent research indicates increased IL-17 expression in cervical cancer. According to the researchers, IL-17 may promote angiogenesis, proliferation, and invasion of cells in this tumor through the NF-*κ*B signalling pathway [15].

Our own research showed significantly higher IL-17A concentration in the plasma of ovarian cancer patients, compared to that in peritoneal fluid. Similar results were obtained by Giuntoli et al., who showed a significant lower concentration of IL-17 in PF, compared to the plasma of patients with advanced ovarian cancer [62]. According to the literature, the source of IL-17 is Th17 cells, as well as monocytes, neutrophils, NK cells, NKT-17, *γδ*-17, CD8^+^ lymphocytes, and epithelial cells [63]. In our own research, we showed a significantly higher percentage of Th17 cells in the peripheral blood of patients with OC than that in PF. This observation indicates more intensified activity of the assessed cell population in the synthesis of IL-17 in the PB of OC patients.

In this context, interesting results were obtained by Kryczek et al., who showed a relationship between the concentration of IL-17 in peritoneal fluid and the survival time of OC patients. In patients whose PF contained high levels of IL-17, the average survival time was 78 months. In turn, patients with low levels of IL-17 in the PF lived shorter (27 months). It should be emphasized that this is the only report demonstrating a positive prognostic value of IL-17 in PF in ovarian cancer patients [18]. The results we obtained confirm the reports by Kryczek et al. We showed that the survival of patients with higher levels of IL-17A in peritoneal fluid was significantly longer than that in patients with lower concentration of IL-17A in PF (median: 36.5 months vs. 27 months). The results of the assessment of the relationship between IL-17 concentration in serum and the survival time of cancer patients are inconclusive. According to some studies, high levels of IL-17 correlated with poor prognosis, as demonstrated in patients with NSCLC, colon cancer, hepatocellular carcinoma, leukaemia, and gastric cancer. Other researchers did not observe any significant relationships or showed a positive relationship between IL-17 concentration and survival, as in the case of acute myeloid leukaemia [19]. Similar to our own study, Winkler et al. showed no significant correlations between IL-17A concentration in serum and survival time of ovarian cancer patients [33]. Using immunohistochemistry, Lan et al. showed a higher level of IL-17 expression in OC tissue, compared to controls. In addition, they showed a correlation between a high level of IL-17 expression and longer progression free survival (PFS) in patients with advanced ovarian cancer. However, no significant differences were found in the overall survival time of OC patients [64]. It is suggested that the reason for the observed discrepancies may be the microenvironment of individual tumors, especially the type of cell population responsible for IL-17A synthesis. Th17 cells may dominate in one environment, and neutrophils or mast cells in another [19].

Kryczek et al. showed a significantly higher concentration of IL-17A in the peritoneal fluid of patients with stage III OC according to FIGO, compared to the group with stage IV clinical cancer [18]. We did not observe such differences in our own research. The concentration of IL-17 we found in both plasma and PF did not differ significantly depending on the clinical stage of the disease according to FIGO, the degree of histological differentiation, and the type of cancer according to Kurman and Shih.

However, what is interesting is the significant differences we found in IL-17A concentration in the PF of premenopausal patients with OC, compared to postmenopausal patients. The concentration of IL-17A in the PF of premenopausal OC patients was significantly higher than that in postmenopausal patients. It is known that ovarian cancer mostly affects patients over 60 years of age. Malutan et al. showed a significantly higher concentration of selected proinflammatory cytokines (IL-1*β*, IL-8, and TNF-*α*) in women after natural and surgical menopause without cancer, compared to the control group [65]. They also evaluated the concentration of IL-17 in women depending on menopausal status. Contrary to our observations, no statistically significant differences in IL-17 levels in pre- and postmenopausal women were found in this study. Currently, research is conducted on female sex hormones and their effect on IL-17 concentration in systemic diseases. Perhaps it will help us understand the role of this cytokine in the pathophysiology of cancer. Interleukin 17 induces inflammation and contributes to joint damage in rheumatoid arthritis. The female sex hormone 17β-estradiol (E2) inhibits experimentally induced arthritis. As the main source of IL-17 is *γδ*T cells, the purpose of this study was to investigate whether E2 affects IL-17^+^*γδ* cells during the development of arthritis in various experimental models. The researchers showed that treatment with E2 reduces the concentration of IL-17^+^*γδ*T cells in joints, but increases the concentration of IL-17^+^*γδ*T cells in draining lymph nodes, suggesting that E2 may prevent migration of IL-17^+^*γδ*T cells from lymph nodes into joints [66].

In our studies, we did not find a statistically significant difference in the percentage of CD4^+^IL-17^+^ cells depending on the menopausal status of the patients. The first studies have been published on the effect of changes in sex hormone levels on the percentage of Th17 in autoimmune diseases. Andersson et al. observed the effect of estradiol on the percentage of Th17 in rheumatoid arthritis. Mice transfected with 17*β*-estradiol (E2) showed a reduction in the severity of arthritis and a lower percentage of Th17 in the joints, compared to the control group. Interestingly, mice treated with E2 showed a higher percentage of Th17 in the lymph nodes in the early stages of the disease, depending on ER*α*. E2 was found to increase the expression of C-C chemokine receptor 6 (CCR6) and the corresponding C-C20 ligand (CCL20) on Th17 cells in lymph nodes. These data suggest that this may be related to the CCR6-CCL20 pathway, which is important for Th17 cell migration [66]. Recent studies have shown a significantly higher concentration of IL-17 in the serum of women with a pregnancy complicated by fetal growth restriction (FGR) and preeclampsia, compared to healthy pregnant women. In conjunction with a reduced TGF-*β* level detected in pregnancy complicated by FGR associated with preeclampsia (PE), it may induce an inflammatory response and, consequently, lead to placental insufficiency [67]. Perhaps the conducted research not only will allow us to understand the role of hormonal balance in the development of autoimmune diseases, but will also open up new opportunities for research on the impact of female sex hormones on the immune system in ovarian cancer [66].

The available literature does not include any studies assessing the occurrence of Th17 cells and IL-17 cells in patients with types I and II ovarian cancer according to Kurman and Shih. According to the latest data, the effects of chemotherapy in patients with type II OC are worse than those in patients with type I ovarian cancer. It is estimated that about 90% of patients with type I OC survive five years, while about 90% of patients with type II cancer die within five years of diagnosis [68]. Although the molecular basis of the OC types according to Kurman and Shih seems to be well characterized [68, 69], little is known about how the immune system works in particular types of cancer. The conducted studies did not show any statistically significant differences in the percentage of Th17 cells and IL-17 levels in the examined environments depending on the type of cancer according to Kurman and Shih. Given the significant relationship between OC type and survival described in the literature, research on a larger number of patients with a particular type of cancer according to Kurman and Shih seems advisable.

## 6. Conclusions

To sum up, the results of our own research and the data quoted from the literature indicate a proinflammatory nature of the ovarian cancer microenvironment (high level of IL-17A in PF and a high percentage of Th17 infiltrating OC). These results suggest that Th17 cells/IL-17A may play a beneficial role in OC immunity. What is interesting, higher IL-17A level in the PF correlated with markedly more favorable clinical outcomes of OC patients.

On the other hand, the percentage of Th17 cells was lower in the PB and PF of ovarian cancer patients in comparison to the group with benign ovarian tumor. It may promote evasion of host immune response by cancer cells. The direct mechanism of Th17 action in OC has not been yet studied and remains unexplained. It seems justified to continue the preliminary research to thoroughly understand the mechanisms that play a key role in the process of infiltration of neighboring OC tissues not only in the context of their significance in the biology of OC, but also in the novel target for cellular immunotherapy.

## Figures and Tables

**Table 1:** The clinical characteristics of ovarian cancer patients.

The chosen clinical data	Ovarian cancer (*n* = 71)
Age (median), years (range)	57 (22–79)

FIGO stage, *n* (%)	
Early (I-II)	25 (35.21%)
I	14 (19.72%)
II	11 (15.49%)
Advanced (III-IV)	46 (64.79%)
III	43 (60.56%)
IV	3 (4.23%)

Degree of histological differentiation, *n* (%)	
Intermediate grade (G2)	35 (49.30%)
Low grade (G3)	36 (50.70%)

The OC classification according Kurman and Shih, *n* (%)	
Type I (serous G2, mucinous, endometrioid)	42 (59.15%)
Type II (serous G3)	26 (40.85%)
Ca125 range, median (U/ml)	435.30 (5.71–23935.36)

**Table 2:** Plasma IL-17A concentration in studied group of patients.

Group of patients	Plasma IL-17A concentration (pg/ml)		
	Median	Minimum	Maximum
Ovarian cancer (*n* = 71)	3.80^*∗*^	0.042	30.00
Benign tumors (*n* = 35)	0.229	0.076	1.696
Control group (*n* = 10)	0.533	0.076	1.295

^*∗*^ *p* < 0.0001 compared to both the group of benign ovarian tumors and the control. Clinical significance of IL-17A in the plasma and peritoneal fluid of OC patients.

## Data Availability

The data used to support the findings of this study are included within the article.
